# Contactin-1 and Neurofascin-155/-186 Are Not Targets of Auto-Antibodies in Multifocal Motor Neuropathy

**DOI:** 10.1371/journal.pone.0134274

**Published:** 2015-07-28

**Authors:** Kathrin Doppler, Luise Appeltshauser, Heidrun H. Krämer, Judy King Man Ng, Edgar Meinl, Carmen Villmann, Peter Brophy, Sulayman D. Dib-Hajj, Stephen G. Waxman, Andreas Weishaupt, Claudia Sommer

**Affiliations:** 1 Department of Neurology, University Hospital Würzburg, Würzburg, Germany; 2 Department of Neurology, University Hospital Gießen, Gießen, Germany; 3 Institute of Clinical Neuroimmunology, Ludwig-Maximilians-University, Munich, Germany; 4 Institute for Clinical Neurobiology, University Hospital Würzburg, Würzburg, Germany; 5 Centre for Neuroregeneration, University of Edinburgh, Edinburgh, United Kingdom; 6 Department of Neurology, Yale University School of Medicine, New Haven, Connecticut, 06510, United States of America; 7 Center of Neuroscience and Regeneration Research, Veterans Affairs Medical Center, West Haven, Connecticut, 06516, United States of America; Charité University Medicine Berlin, GERMANY

## Abstract

Multifocal motor neuropathy is an immune mediated disease presenting with multifocal muscle weakness and conduction block. IgM auto-antibodies against the ganglioside GM1 are detectable in about 50% of the patients. Auto-antibodies against the paranodal proteins contactin-1 and neurofascin-155 and the nodal protein neurofascin-186 have been detected in subgroups of patients with chronic inflammatory demyelinating polyneuropathy. Recently, auto-antibodies against neurofascin-186 and gliomedin were described in more than 60% of patients with multifocal motor neuropathy. In the current study, we aimed to validate this finding, using a combination of different assays for auto-antibody detection. In addition we intended to detect further auto-antibodies against paranodal proteins, specifically contactin-1 and neurofascin-155 in multifocal motor neuropathy patients’ sera. We analyzed sera of 33 patients with well-characterized multifocal motor neuropathy for IgM or IgG anti-contactin-1, anti-neurofascin-155 or -186 antibodies using enzyme-linked immunosorbent assay, binding assays with transfected human embryonic kidney 293 cells and murine teased fibers. We did not detect any IgM or IgG auto-antibodies against contactin-1, neurofascin-155 or -186 in any of our multifocal motor neuropathy patients. We conclude that auto-antibodies against contactin-1, neurofascin-155 and -186 do not play a relevant role in the pathogenesis in this cohort with multifocal motor neuropathy.

## Introduction

Multifocal motor neuropathy (MMN) is a rare, chronic-progressive disorder affecting peripheral motor nerves, leading to asymmetric weakness of limbs, often most pronounced in the distal and middle segments of the upper extremities [1]. Diagnostic criteria are based on clinical symptoms and conduction block in nerve conduction studies outside common nerve compression sites [1]. Although details of the exact pathophysiology of the disease need to be further elucidated, a role of the immune system can be assumed since IgM anti-GM1 antibodies are detected in about half of all MMN patients [2] and since the disease responds to treatment with high-dose intravenous immunoglobulins (IVIG). Other anti-ganglioside antibodies are only found in few percent of patients [3]. This implies that, in almost half of all patients with MMN, an associated auto-antibody cannot be detected. Several recent studies have focused on the detection of auto-antibodies against proteins of the paranodal and nodal complexes [4,5,6,7] that are cell adhesion molecules and form a link between the myelin sheath and the axon and contribute to the assembly of ion channels that are essential for saltatory nerve conduction. Antibodies against contactin-1, neurofascin and gliomedin have been reported to be present in 2–10% of patients with chronic inflammatory demyelinating polyneuropathy (CIDP) and are supposed to be associated with a typical clinical phenotype of acute onset severe sensorimotor neuropathy and tremor [4,5,6,7]. Antibodies against neurofascin lead to conduction block after intraneural injection into rat sciatic nerves [8], suggesting that these proteins may potentially be targets in MMN as well. However, the clinical phenotype of MMN patients is completely different compared to patients with anti-contactin-1 or anti-neurofascin auto-antibodies. A previous study by Notturno and coworkers reported that auto-antibodies against neurofascin-186 and gliomedin could be detected in 62% of patients with MMN by ELISA [9,10]. These findings are of great interest, as diagnosis of MMN is often challenging in clinical practice and valid biomarkers are urgently needed. In the present study, we aimed to validate and extend these finding, by determining the frequency of detecting auto-antibodies against neurofascin-186 and the paranodal proteins neurofascin-155 and contactin-1 using three different detection assays including ELISA, cell binding assays, binding assays with teased fibers.

## Subjects and Methods

### Ethics statement

The study was approved by the ethics committee of the Medical Faculty of the University of Würzburg and was performed in accordance with the ethical standards of the Declaration of Helsinki of 1964. All patients and controls gave written informed consent to take part in the study.

### Patients

A total number of 33 patients with MMN attending the Departments of Neurology of the University Hospitals Würzburg and Gießen were prospectively recruited in 2013 and 2014. Diagnosis was based on the EFNS criteria [1]: 25 patients were classified as definite MMN, two as probable MMN and six as possible MMN. Sera were obtained prior to the initiation of treatment in five patients, during a period without treatment in two patients and under immunoglobulin treatment in all other cases. In the latter cases, sera were obtained immediately before application of IVIG, with an interval of at least two weeks from the last application, except for two patients with subcutaneous application of immunoglobulins. Sixty sera of healthy individuals (mean age 54.4 years, 31 males, 29 females) and 10 sera of patients with other autoimmune neurological diseases (myasthenia gravis, multiple sclerosis; mean age 56.9 years, 3 males, 7 females) served as controls. Sera of CIDP patients with anti-contactin-1 and anti-neurofascin-155 auto-antibodies described in previous studies were used as positive controls [6,11]. Routine work-up of the MMN patients included clinical examination by neurologists of the Departments of Neurology of the University Hospitals Würzburg and Gießen and nerve conduction studies according to the EFNS guidelines [1]. Muscle strength was quantified using the Medical Research Council (MRC) scale [12] and weakness was determined as a score of 4 or less. All sera were tested for anti-ganglioside antibodies (GM1 IgM/IgG, GM2 IgM/IgG, GM3 IgM/IgG, GD1a IgM/IgG, GD1b IgM/IgG, GT1b IgM/IgG, GQ1b IgM/IgG; Euroimmun, Lübeck, Germany).

### Binding assays with murine teased fibers

Sciatic nerves were removed from adult female C57BL/6 mice and were pre-fixed in 4% paraformaldehyde, and teased fibers were prepared by gently teasing the single nerve fibers with two fine forceps on a glass slide. Teased fiber preparations were air-dried overnight, fixed with acetone and incubated with blocking solution containing 4% normal goat serum, 4% fetal calf serum, 0.3% Triton-X-100 in PBS. The preparations were incubated with patients’ sera diluted 1:100 overnight at 4°C following incubation with appropriate anti-human Cy3-conjugated secondary antibodies (Dianova, Hamburg, Germany, 1:100) for two hours at room temperature. Double-immunofluorescence staining using patients’ sera and polyclonal rabbit anti-caspr (Abcam, Cambridge, UK, 1:1,000) or polyclonal rabbit anti-pan-neurofascin (Abcam, 1:400) was performed to detect paranodes. Sera of patients with anti-contactin-1-IgG-positive CIDP were used as a positive control. They were mounted with VECTASHIELD with DAPI (Vector Laboratories, Burlingame, CA, USA). Slides were assessed using a fluorescence microscope (Zeiss Ax10, Zeiss, Oberkochen, Germany). The examiner was blinded to the diagnosis and clinical data of the patients.

### Enzyme-linked immunosorbent assay (ELISA)

Maxisorb 96-well-plates (Thermo Fisher Scientific, Waltham, MA, USA) were coated with human contactin-1 (Sino Biological, Beijing, China) diluted at a concentration of 2 μg/ml in 0.1 M carbonate buffer (pH 9.6) and incubated at 4°C overnight. Neurofascin ELISA was performed as previously described using full-length human neurofascin-155 and -186 [6]. All wells were washed with PBS/0.05% Tween-20 and blocked with PBS/3%BSA/0.05% Tween-20 for one hour at 37°C, the wells were then incubated with patients’ samples (1:100) or anti-contactin-1 (mouse monoclonal, Abcam, 1:200) or anti-neurofascin-155 (rabbit anti-neurofascin 155 raised against a peptide derived from the Fibronectin III C domain unique to NF155; 1:200) [13] or anti-neurofascin-186 (rabbit anti-NF186 raised to a peptide derived from the Mucin domain unique to NF186, 1:1000)[13] for 1 h at 37°C. After washing with PBS/0.05% Tween-20, the plate was incubated with appropriate horseradish-conjugated secondary antibodies (anti-human-IgG or IgM for patients material, 1:10,000/1:7,000, DakoCytomation, Glostrup, Denmark; anti-mouse/-rabbit IgG for the control antibody, 1:500). 100 μl of TMB solution (eBioscience, San Diego, CA, USA) were added to each well and the reaction was stopped after fifteen minutes by addition of 50 μl of 1M sulfuric acid. Optical density was measured at 450 nm with Multiscan EX Elisa Reader (Thermo Fisher Scientific, Waltham, MA, USA). Sera of CIDP patients with anti-contactin 1 IgG and anti-neurofascin-155 IgG/IgM were used as controls. Normal controls were run in each assay. The normal value was set at five standard deviations above the mean of all healthy controls, based on the distribution of values of normal controls and of anti-contactin-1-positive/anti-neurofascin positive CIDP patients. IgM and IgG serum levels were determined by human IgM and IgG ELISA kits (AssayPro, St. Charles, MO, USA) according to the instructions of the manufacturer.

### Binding assays with transfected human embryonic kidney (HEK) 293 cells

HEK293 cells were plated onto poly-D-lysine-coated cover slips in a 24-well-plate at a density of 90,000 cells/well. HEK293 cells were transiently transfected with plasmids of rat contactin-1 [14], human neurofascin-155 and -186 [6] using the calcium-phosphate precipitation method (0.25 μg of DNA/well).[15] Rat contactin-1 is 95% identical to human contactin-1 at the amino acid level, thus rat-contactin-1 is suitable in this assay. With this protocol about 20% of all cells expressed the respective protein on their surface. 48 hours after transfection, cells were fixed with 4% paraformaldehyde and were incubated with patients’ sera (1:500) and anti-contactin-1 (Abcam, 1:200) or anti-neurofascin 155 and 186 (1:100) as a control antibody and appropriate secondary antibodies (Cy3 and AlexaFluor-conjugated anti-human IgG or anti-mouse IgG). Sera of patients with anti-contactin-1-positive CIDP were used as a positive control. Immunostaining was assessed using a fluorescence microscope (Zeiss Ax10, see above).

## Results

### Patients

Thirty-three patients with a diagnosis of MMN were included (mean age 57.1 years, median 57 years, range 33–76 years, 23 men, 10 women), see also Table 1. Disease duration ranged between 0.5 and 27 years, with a mean of 8.8 years and a median of 9 years. Patients were recruited at the University Hospitals of Würzburg (n = 23) and Gießen (n = 10). The majority of patients showed the typical clinical picture of multifocal motor neuropathy with muscle weakness beginning at the hand muscles, spreading to more proximal muscles and to the lower limbs, and muscle atrophy during the course of disease. Onset of muscle weakness at the lower limbs was reported by four patients and remained restricted to the lower limbs in three patients. None of the patients reported any relevant sensory deficits. Conduction blocks were detected in all patients except for five individuals that were classified as possible MMN. All patients had a good response to immunoglobulin treatment.

**Table 1:** Summary of clinical data of the MMN cohort. CSF = cerebrospinal fluid.

	Number of patients (total number n = 33)
Median age (years) (range)	57 (33–76)
Sex	23 men, 10 women
Median duration of disease (years) (range)	9 (0.5–27)
Muscle weakness	Upper limbs: 10, lower limbs: 3, both: 20
Decreased/absent deep tendon reflexes	25
Muscular atrophy	21
Elevated CSF protein	5
Conduction block	28

Anti-ganglioside auto-antibodies were tested in all patients and GM1 IgM auto-antibodies were detected in 14/33 patients. GM2 IgM auto-antibodies were found in 5/33 patients. IgM auto-antibodies against GM2, GD1b, GT1b and GQ1b were found in one patient.

IgG levels were between 17.91 mg/ml and 40.61 mg/ml with a median of 29.76 mg/ml in MMN patients, between 8.84 mg/ml and 24.56 mg/ml in healthy controls (median 15.86 mg/ml) and between 5.47 mg/ml and 23.52 mg/ml (median 11.69 mg/ml) in disease controls. IgM levels ranged from 1.14 mg/ml to 6.3 mg/ml with a median of 3.94 mg/ml in MMN patients, from 1.19 mg/ml and 2.63 mg/ml in healthy controls (median 1.68 mg/ml) and from 1.53 mg/ml to 2.95 mg/ml (median 2.19 mg/ml) in disease controls. Thus IgG and IgM levels were elevated in patients with MMN compared to controls, which is most likely a consequence of the patients’ regular IVIG treatment.

### ELISA

**Fig 1:**
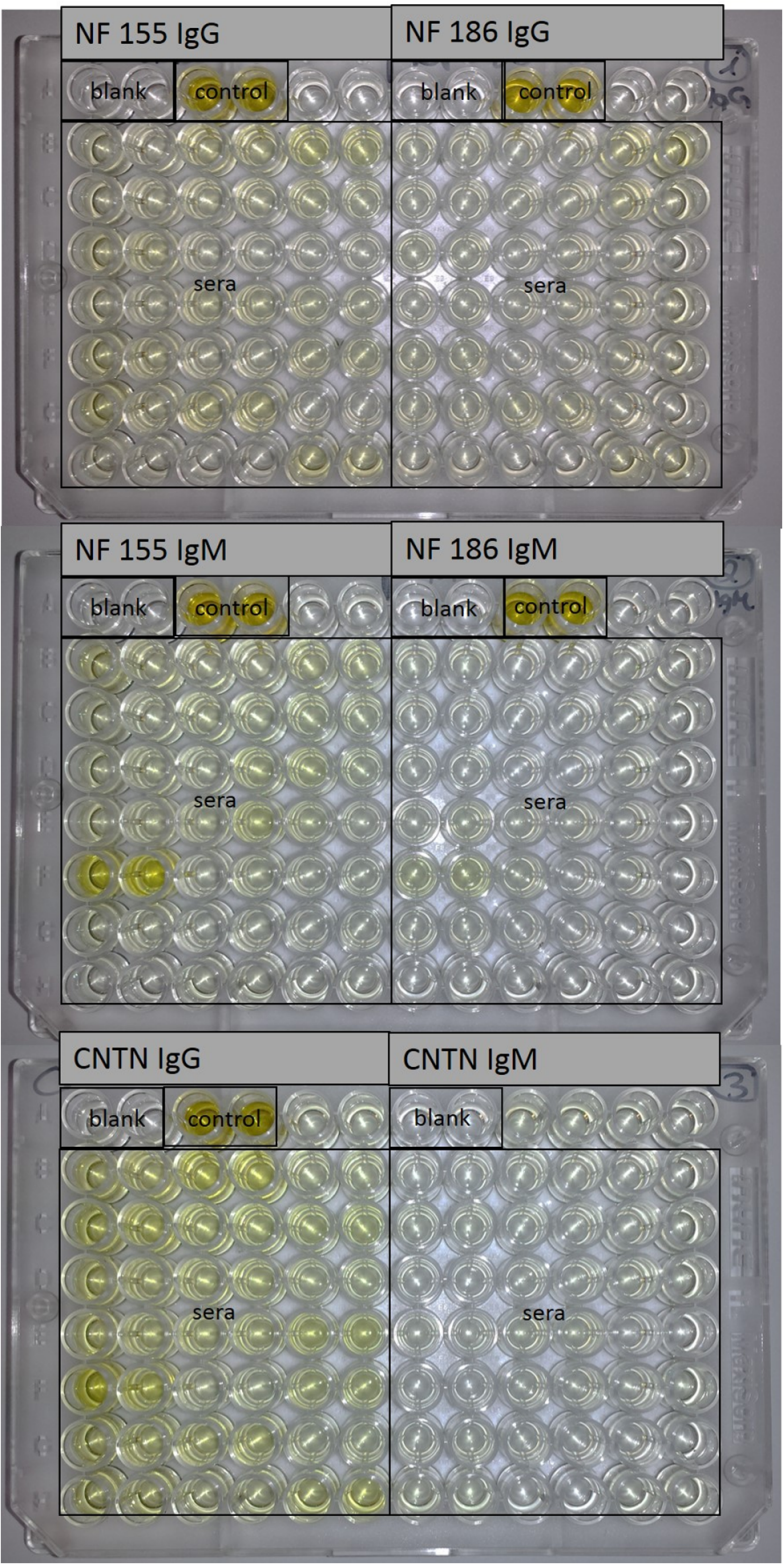
ELISA plates. Pictures of the ELISA plates with assays for neurofascin-155, neurofascin-186 and contactin-1. Controls show high optical density in contrast to sera.

**Fig 2:**
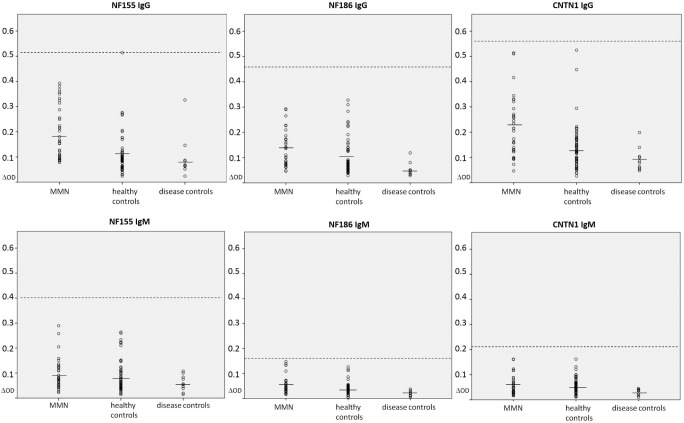
Dot plots showing the optical density measured by ELISA. Samples from patients with MMN, healthy and disease controls reacting to neurofascin-155 IgG (A), neurofascin-186 IgG (B), contactin-1-IgG (C), neurofascin-155 IgM (D), neurofascin-186 IgM (E) and contactin-1 IgM (F) are shown. All patients except for one control in the neurofascin-155 IgG assay are within normal range (dashed line, five standard deviations above the mean of all controls (line)). OD = optical density, NF = neurofascin, CNTN = contactin

ELISA was performed using sera from all patients and controls. Antibodies against contactin-1, neurofascin-155 and -186 served as positive controls in each assay and showed a clear immunoreaction (Fig 1). All three ELISA protocols had been tested in previous studies, demonstrating efficacy and specificity to detecting the respective auto-antibodies [6]. We did not detect any IgM or IgG auto-antibodies against neurofasin-155, -186 or contactin-1 in any patient with multifocal motor neuropathy. (Fig 2A–2F). Slightly elevated IgG reaction to neurofascin-155 was found by ELISA in one healthy control (Fig 2A).

### Cell binding assays

**Fig 3:**
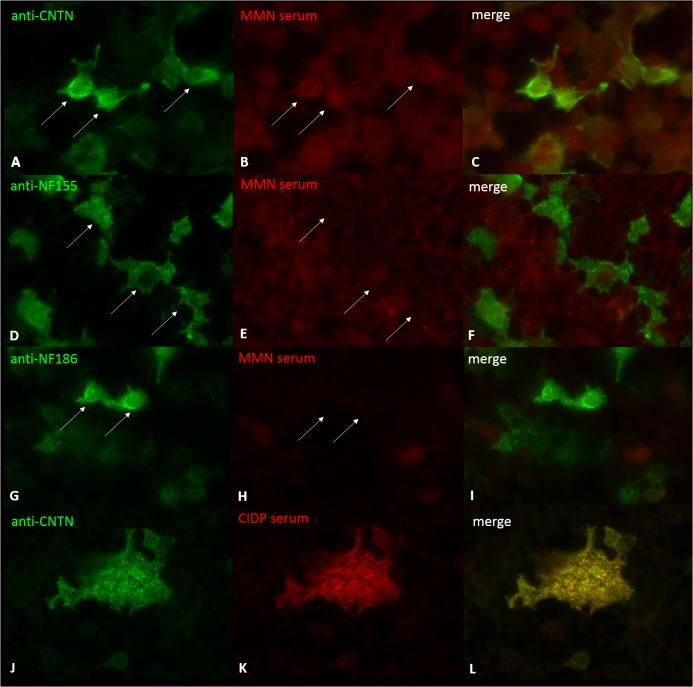
Immunofluorescence of transfected HEK293 cells. Double-immunofluorescence of contactin-1-, neurofascin-155- and neurofascin-186-transfected HEK293 cells with a control anti-contactin antibody (A, J), control anti-neurofascin155 (D), control anti-neurofascin186 (G) and sera of patients with MMN (B, E, H) and serum of an anti-contactin-1-positive CIDP control (K). Transfected cells (arrows) are stained with anti-contactin-1 (A), anti-neurofascin (D, G), and with the serum of the anti-contactin-1-positive CIDP patient (K) but not with MMN patients’ sera (B, E, H).

Cell binding assays were performed with all sera. Antibodies against neurofascin-155, -186 and contactin-1 served as positive controls and showed good binding to transfected cells (Fig 3A, 3D and 3G). However, we did not detect any binding of patients’ or controls’ sera to HEK293 cells transfected with contactin-1, neurofascin-155 or -186 (Fig 3B, 3E and 3H).

### Binding assays with murine teased fibers

**Fig 4:**
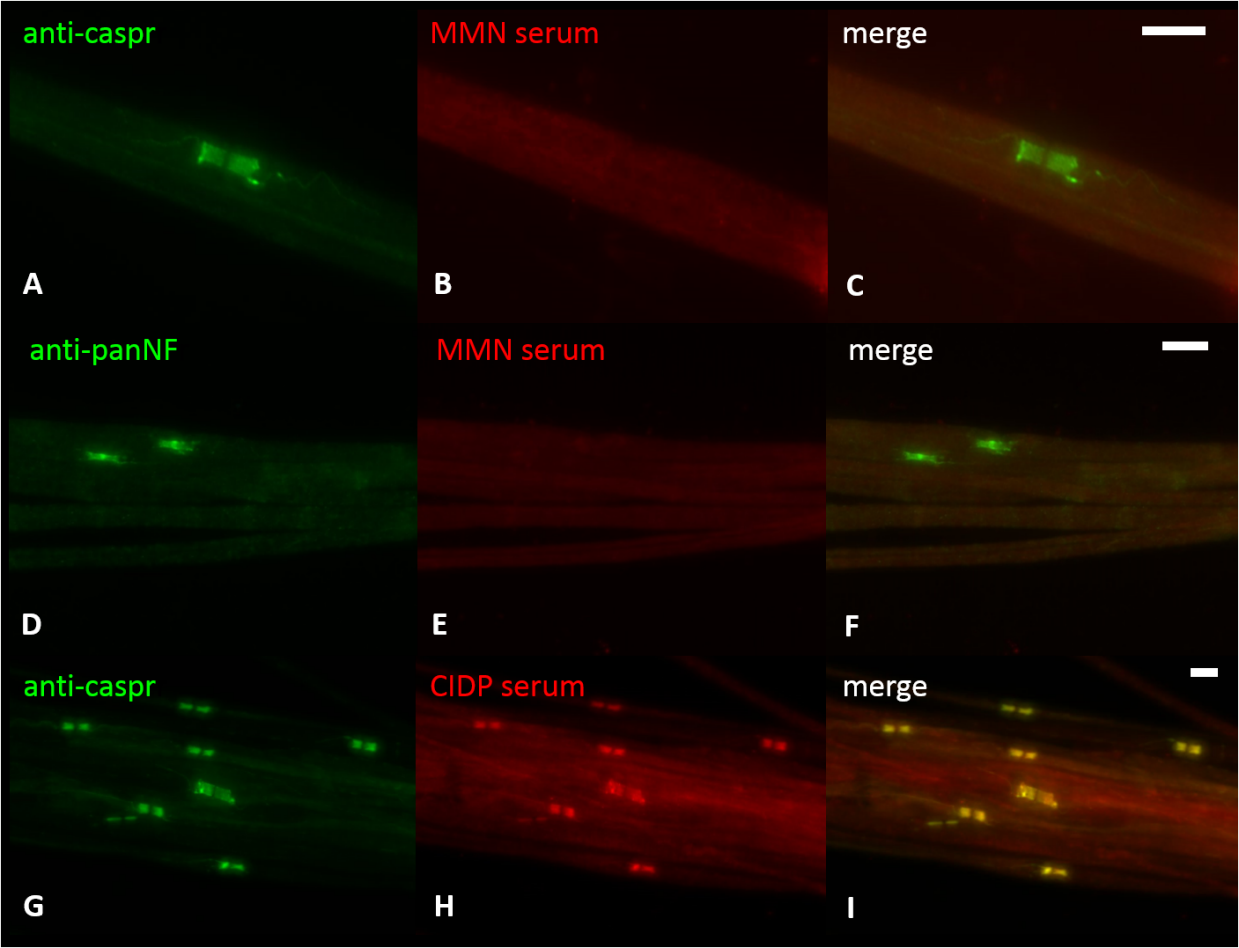
Binding assays with murine teased fibers. Double-immunofluorescence of murine teased fibers with anti-caspr as a paranodal marker (A, G) and anti-pan-neurofascin (D) and sera of patients with MMN (B, E) and serum of a patient with anti-contactin-1-positive CIDP (H). No colocalization of binding of MMN patients’ sera and caspr or pan-neurofascin can be detected. Clear colocalization of anti-caspr and patient’s serum can be seen in the CIDP control (G-I). Scale bar = 5 μm.

We did not detect any specific binding of patients’ sera to the paranodal or nodal region of murine teased fibers resembling the binding pattern of anti-caspr as a paranodal marker (Fig 4A–4C) or anti-neurofascin-155 or -186 (Fig 4D–4F).

## Discussion

In the present study, we did not detect auto-antibodies against contactin-1, neurofascin-155 or -186 in a cohort of 33 patients with MMN using ELISA with human neurofascin-155, -186 and contactin-1 and cell binding assays with transfected HEK293 cells and did not identify any binding of sera to murine teased fibers colocalizing with markers of the paranodal or nodal region.

Our results are in contrast to a recent study, reporting auto-antibodies against neurofascin-186 in more than 60% of patients with MMN using ELISA with a rat recombinant neurofascin-186 peptide [9]. However, there are some differences between the two studies. In the study by Notturno et al., a rat neurofascin-186 peptide was used for the ELISA while we used human full-length proteins where the overall fold should be similar to native protein and epitopes should be received. Furthermore, detection of auto-antibodies was solely based on ELISA in their study, and binding assays using transfected HEK293 cells were negative in all patients which corresponds to our results. We additionally included binding assays with murine teased fibers. The discrepancy of results in the ELISA may be explained by the use of different proteins. Ng et al. have recently compared ELISA with different proteins (human neurofascin-186 and -155 vs. rat neurofascin-155) and found considerable differences in the specificity of antibody binding probably due to abnormally glycosylated immunogenic proteins produced in NS0 myeloma cell lines [6]. Differences between patient cohorts are unlikely to be the cause of the discrepant findings as multifocal motor neuropathy is a well-defined disease, patients fulfilling similar diagnostic criteria were included in the studies, and both cohorts included Caucasian patients.

It could be argued that ongoing IVIG treatment in our patients might interfere with the presence of auto-antibodies against contactin-1 and neurofascin-155 or -186. However, titers of IgM GM1 antibodies are hardly influenced by treatment with IVIG in MMN patients [16] and titers of other auto-antibodies in neuromuscular diseases have been shown to decrease but remain detectable under treatment with IVIG [17]. Moreover, the patients reported by Notturno and coworkers were not all treatment-naïve, some were under IVIG treatment, although the exact numbers were not provided [10].

Recent studies have described a uniform phenotype of patients with anti-neurofascin and anti-contactin-1 auto-antibodies with acute GBS-like onset of sensorimotor neuropathy following chronic course of disease and severe tremor in patients with auto-antibodies against neurofascin-155 [4,5]. Our patients, in contrast, showed the typical clinical picture of MMN with chronically progressive multifocal weakness without relevant sensory loss.

We therefore conclude that anti-neurofascin and anti-contactin auto-antibodies are not detectable in our cohort of patients with MMN, suggesting that these auto-antibodies are probably not pathogenetically relevant in MMN. Considering the contrasting findings with the study by Notturno et al. that showed positive results using an ELISA with a rat neurofascin-186 peptide, it needs to be taken into account that confirmatory tests and the use of full-length proteins may be useful for a reliable result of antibody detection. Our study does not provide any evidence that contactin-1 or neurofascin-155/186 are relevant targets in MMN and the fundamental question of what might be targeted in MMN remains to be resolved.
