# Correction: Contactin-1 and Neurofascin-155/-186 Are Not Targets of Auto-Antibodies in Multifocal Motor Neuropathy

**DOI:** 10.1371/journal.pone.0137443

**Published:** 2015-08-28

**Authors:** Kathrin Doppler, Luise Appeltshauser, Heidrun H. Krämer, Judy King Man Ng, Edgar Meinl, Carmen Villmann, Peter Brophy, Sulayman D. Dib-Hajj, Stephen G. Waxman, Andreas Weishaupt, Claudia Sommer

The following information is missing from the Funding section: This publication was funded by the German Research Foundation (DFG) and the University of Wuerzburg in the funding programme Open Access Publishing.

## References

[1] Doppler K, Appeltshauser L, Krämer HH, Ng JKM, Meinl E, Villmann C, et al (2015) Contactin-1 and Neurofascin-155/-186 Are Not Targets of Auto-Antibodies in Multifocal Motor Neuropathy. PLoS ONE 10(7): e0134274 doi: 10.1371/journal.pone.0134274 26218529 10.1371/journal.pone.0134274 PMC4517860

